# Frailty in Older Adults

**DOI:** 10.1093/geroni/igaf122.1087

**Published:** 2025-12-31

## Abstract

The assessment, trajectory and biomarkers of frailty in older adults will be presented.

